# P21参与肺癌耐药的研究进展

**DOI:** 10.3779/j.issn.1009-3419.2020.101.16

**Published:** 2020-07-20

**Authors:** 甜 符, 爱玲 梁, 勇军 刘

**Affiliations:** 1 523808 东莞，广东省医学分子诊断重点实验室 Medical Molecular Diagnostics Key Laboratory of Guangdong, Dongguan 523808, China; 2 523808 东莞, 广东医科大学基础医学院生物化学与分子生物学教研室 Department of Biochemistry and Molecular Biology and Department of Clinical Biochemistry in Guangdong Medical University, Dongguan 523808, China; 3 523808 东莞, 广东医科大学医学检验学院临床生物化学教研室 Department of Clinical Laboratory Biochemistry of Guangdong Medical University, Dongguan 523808, China

**Keywords:** 肺肿瘤, P21, 耐药, Lung neoplasms, P21, Drug resistance

## Abstract

肺癌是全球发生率最高和死亡人数最多的恶性肿瘤。近些年肺癌的治疗取得重大突破，但是随着病情的进展，患者不可避免地出现耐药，药物疗效大大降低。P21蛋白是一种在肿瘤中发挥双重作用的蛋白，既能调控细胞周期、诱导细胞凋亡、抑制细胞增殖，也能保护细胞抵抗凋亡，在肿瘤细胞耐药中发挥重要作用。本文就P21与肺癌耐药及其相关研究进行综述，为临床肺癌的个体化治疗与克服肺癌耐药提供新的思路。

## Abstract (en)

Lung cancer is the most common malignant tumor in the world with the highest incidence of deaths. In recent years, the treatment of lung cancer has made a significant breakthrough. However, as the tumor progresses, lung cancer cells inevitably acquire resistance and the efficacy of the treatment are greatly reduced. P21 protein plays a dual role in tumors, which not only regulates the cell cycle, induces apoptosis, inhibits cell proliferation, but also protects cells against apoptosis and promotes tumor cell resistance. This article reviews the research on P21 and lung cancer resistance, to provide new ideas for individualized treatment of lung cancer and overcoming lung cancer resistance.

根据2018年全球癌症统计数据^[1]^显示，2018年全球肺癌新增病例数占总癌症病例数的11.6%，其中肺癌死亡人数占癌症总死亡人数的18.4%，是全球人类发生率最高和导致癌症死亡的主要原因。手术是早期肺癌患者的主要治疗手段，但是由于肺癌患者早期症状不明显，发病隐匿，大部分患者确诊时往往已是中晚期。放疗、化疗、靶向治疗和免疫治疗是中晚期肺癌患者的主要治疗方法，靶向治疗是近十几年来肺癌治疗取得的巨大突破，免疫治疗是近几年肺癌治疗的新方法。尽管这些不同的治疗方法有较好的疗效，能有效缓解患者痛苦并提高生存期，但是随着病情的进展，患者在用药一段时间后仍不可避免地出现获得性耐药，获得性耐药是导致临床肺癌患者治疗失败和癌症治疗最难以克服的问题之一。因此，深入探讨肺癌耐药机制、逆转肺癌耐药是研究肺癌的难点和热点。

P21又叫P21^Waf1/Cip1^或CDKN1A（cyclin-dependent kinase inhibitor 1A），最先被el-Deiry等^[2]^运用差减杂交技术发现，它处于野生型*p53*的下游，是由野生型*p53*活化片段基因1（wild-type p53-activated fragment, WAF1）编码的大小为21 kDa的蛋白质。el-Deiry等^[2]^发现*p53*能直接诱导*p21*的表达并且它可能是P53发挥抑癌作用的中介。与此同时，Harper等^[3]^利用酵母双杂交系统发现了能编码21 kDa的细胞周期依赖性激酶（cyclin-dependent protein, CDK）相互作用蛋白的基因，并将其命名为细胞周期依赖性激酶相互作用蛋白1（CDK interaction protein 1, CIP1）；该蛋白通过与CDKs紧密结合，从而在细胞周期中发挥负性调控作用。然而，P21还在保护细胞抵抗凋亡方面发挥着重要作用，因此P21在肿瘤的发生、发展以及肿瘤药物治疗的敏感性中也发挥着重要作用^[4]^。本文就P21与肺癌耐药及其相关研究进行综述。

## 1 P21蛋白的生物学功能

P21最先作为细胞周期负性调节因子被发现，与P53构成细胞周期检查点，当DNA发生损伤时，P21被激活使细胞周期阻滞直至DNA修复完成，因此它在诱导细胞衰老、凋亡、促进DNA修复、维持基因组的稳定性等方面起重要作用^[5-7]^。然而，P21也能促进肿瘤细胞侵袭、抵抗凋亡，在肿瘤细胞耐药方面发挥着不可忽视的作用^[8, 9]^。

## 2 P21的调控

### 2.1 P53依赖途径

*p53*是*p21*的主要转录调控因子，P21最初被认为是一种肿瘤抑制因子，是DNA损伤诱导的P53依赖细胞周期阻滞的主要介质。P21可以与P53构成细胞周期G_1_期检查点，如图 1所示，当DNA损伤或在其他应激条件下，*p53*被激活，直接与*p21*启动子结合而激活其表达，P21蛋白通过与CDK4、CDK6/cyclin D或CDK2/cyclin E结合，从而抑制细胞周期蛋白依赖激酶活性，使视网膜细胞瘤（retinoblastoma, Rb）蛋白不能被磷酸化，从而可以与转录因子E2F牢固结合而抑制E2F的活性，阻断DNA复制，使细胞周期阻滞直至修复完成，这样能减少受损DNA的复制和积累，阻止异常细胞的增殖以维持基因的保真度从而发挥抑癌作用^[10]^。P21主要通过阻滞细胞周期发挥抑癌作用，大部分研究表明，抑制P21表达能促进肿瘤的发生发展^[11-14]^，诱导P21表达能抑制肿瘤细胞的增殖^[15]^。

**1:**
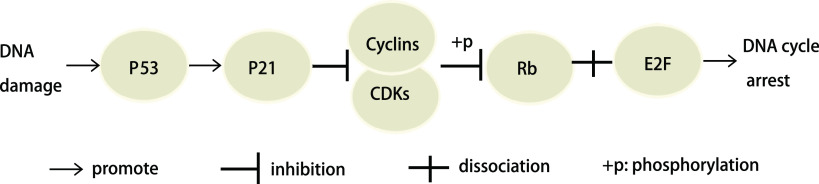
P21阻滞细胞周期图 P21 involved in blocking the cell cycle

此外，P53不仅能直接激活P21的表达发挥抑癌作用，还能通过直接与其形成复合物而发挥作用。Kim等^[16]^的研究结果首次阐明P53和P21共同作用的抑癌机制：P53、P21能结合Mdm2及其底物锌指转录因子Slug蛋白，形成P53/P21/Mdm2/Slug复合物，促进Mdm2依赖的Slug泛素化导致其降解，由于Slug能增强肿瘤细胞的侵袭性，因此Slug泛素化过程可能有助于抑制肿瘤细胞侵袭。除此之外，P53、P21能分别与Bcl-2家族蛋白（例如Bcl-w、Bcl-XL）结合，通过形成P53/P21/Bcl-w复合物使Bax蛋白释放，促进肿瘤细胞凋亡，从而抑制肿瘤细胞的侵袭^[17]^。

### 2.2 P53非依赖途径

P21复杂的调控途径是使其在肿瘤中发挥双重作用的基础。P21还可以通过非P53依赖途径被调控，比如：c-Myc可以直接与P21的羧基端结合，影响P21与PCNA的相互作用，降低了P21介导的DNA合成抑制作用^[18]^。SP1蛋白在*p21*启动子的近端与启动子富含GC的模体结合，对*p21*转录也至关重要^[19]^。信号传导及转录激活蛋白（signal transducer and activator of transcription, STAT）能介导P21的转录、表达和核定位，从而抑制耐药乳腺癌细胞的增殖和侵袭^[20]^。

## 3 P21与肿瘤耐药

P21在肿瘤中具有双重的作用，它的作用主要取决于细胞状态及其在细胞内的定位。2017年Manu等^[21]^研究发现抑制异丙氨酸半胱氨酸羧甲基转移酶能促进P21表达，P21表达上调诱导细胞周期阻滞及激活促凋亡蛋白BNIP3（BCL2/adenovirus E1B 19 kDa interacting protein 3）诱导细胞凋亡，从而增加胰腺癌对药物治疗的敏感性。野生型*p53*的存在是P21发挥抑癌作用的关键；如果*p53*发生突变，P21过表达会解除复制许可，细胞增殖会增加，表现出一定耐药倾向。2016年Galanos等^[22]^在体内与体外实验的研究中发现，在*p53*突变的肿瘤中，长期诱导P21过表达的细胞中，部分肿瘤细胞“躲避”了P21诱导的周期阻滞和细胞衰老，重新进入细胞周期，而且这些细胞中出现广泛的DNA修复，具有更强的侵袭性，且对阿霉素、顺铂等药物有更高的耐受能力。这表明在*p53*突变后，P21过表达可使DNA修复出错和基因组不稳定而导致肿瘤细胞更强的侵袭性和耐药能力。因此突变型*p53*可能是*p21*由抑癌基因转化成致癌基因的关键因素。

目前普遍认为胞核P21主要作用于细胞周期，抑制细胞增殖和促进凋亡，而胞质P21能保护肿瘤细胞免受凋亡，与肿瘤细胞的耐药相关^[23, 24]^。2014年Huang等^[25]^研究发现胞质和胞核P21在胃癌的发展、分期、预后等中发挥相反的作用，胞核P21抑制而胞质P21促进细胞迁移和侵袭能力。2016年Xie等^[26]^研究发现在浸润性肥大细胞介导多西他赛耐药的前列腺癌细胞中，通过调节P38/P53信号通路，使P21的总表达水平及胞质水平均升高；再通过敲减P21能逆转其耐药。但文中未能阐明其机制。2018年Maiuthed等^[27]^证明了在5-氟尿嘧啶耐药的结直肠癌细胞中，胞质P21通过将促凋亡蛋白p-Chk2从细胞核中释放到胞质，使p-Chk2下游的促凋亡蛋白E2F1的激活减少，从而保护肿瘤细胞免受凋亡，介导结肠癌细胞对5-氟尿嘧啶耐药。

P21是一种核蛋白，它能否在胞质大量累积取决于激酶在其不同位点的磷酸化，例如Akt能通过PI3K/Akt信号通路使P21磷酸化导致其在胞质中积累^[28]^。P21在不同情况下发挥相反的作用可能是由于P21不同的细胞内定位造成的。胞核P21能阻滞细胞周期、促进细胞凋亡，而胞质P21能通过降低凋亡蛋白活性或抑制凋亡信号通路，促进肿瘤细胞增殖，保护肿瘤细胞抵抗凋亡，其表达和细胞内定位有望作为肿瘤药物治疗敏感性的判断指标^[24, 25, 28]^。

## 4 P21与肺癌耐药

P21表达水平与肺癌细胞增殖：P21的表达水平及其在细胞内分布与其功能密切相关。2017年Li等^[29]^研究发现miR-1236-3p和miR-370-5p可促进P21的表达，使E-cadherin表达水平上调，同时抑制cyclin D1-CDK4/CDK6，从而诱导细胞周期阻滞、细胞衰老并抑制肺癌细胞的增殖、迁移和侵袭。2019年Guo等^[30]^研究发现A549细胞中抑制长非编码RNA DANCR的水平能促进P21的表达水平增加，使细胞周期阻滞在G_1_期，从而抑制肺癌细胞的增殖。这说明P21表达水平与肺癌细胞增殖呈负相关。然而，2018年Su等^[31]^研究发现敲减肺癌细胞的长非编码RNA MIR22HG后，P21表达水平升高且大部分位于细胞质，同时促进了肺癌细胞增殖。经过转染P21 siRNA处理后，肺癌细胞增殖减少。这些结果表明，P21水平升高促进了肺癌细胞增殖，它的水平与肺癌细胞增殖呈正相关。

P21与肺癌耐药：P21表达水平与肺癌化疗、靶向治疗、放疗的敏感性相关，主要通过调节细胞周期参与肺癌耐药。在不同的研究发现中，其作用和机制如表 1所示^[32-43]^。P21的表达水平下降介导耐药。顺铂是细胞周期非特异性药物，是肺癌化疗的常用药物。2013年Liu等^[32]^研究认为上调P21表达水平能使肺癌细胞周期阻滞，抑制细胞增殖，增加肺腺细胞对顺铂敏感性。有研究^[33]^发现miR-224靶向P21使其表达水平降低，介导A549细胞对顺铂耐药。在耐药细胞中过表达P21后，不仅使细胞周期阻滞在G_1_期/S期，还降低抗凋亡蛋白Bcl-2、Bcl-xL的表达，促进促凋亡蛋白Bax、Bak的表达，因而促进细胞凋亡从而逆转顺铂耐药。Feng等^[34]^研究表明在顺铂耐药的A549细胞中，过表达膜联蛋白A2可激活JNK/c-Jun信号通路抑制*p53*的表达，进而使*p21*等凋亡相关基因表达下降，抑制顺铂诱导细胞凋亡，从而增强细胞对顺铂的耐受性。P21不仅参与了肺癌细胞对顺铂产生耐药，也与其他药物化疗耐药相关。2016年Shan等^[35]^的研究发现在耐甲氨蝶呤的A549细胞中，过表达miR-200c能激活P53/P21信号通路来诱导细胞周期阻滞在G_0_期/G_1_期及诱导细胞凋亡，从而逆转耐药。在Noro等^[36]^的研究中，药物作用使*p21*启动子组蛋白乙酰化能诱导*p21*的表达来阻断Rb-E2F1通路，从而增加肺癌细胞对5-氟尿嘧啶的敏感性。这些结果提示，P21主要通过调控不同期的细胞周期阻滞介导肺癌细胞对化疗药物治疗的敏感性。

**1:** P21在肺癌耐药中的作用及相关机制 The role of P21 in lung cancer resistance and related mechanisms

Type of cells	Type of resistance	Effect of P21	Gene/Pathway interation	References
A549	Cisplatin	Sensitive	lnc RNA HOTAIR	[32]
A549	Cisplatin	Sensitive	MiR-224	[33]
A549	Cisplatin	Sensitive	MiR-33p-3b	[43]
A549	Cisplatin	Sensitive	Annexin A2, JNK/c-Jun, p53	[34]
A549	Methotrexate	Sensitive	miR-200c, P53	[35]
PC9/f14	5-fluorouracil	Sensitive	Rb-E2F1, p53	[36]
PC-9, H1299	Gefitinib	Sensitive	Cyclins-CDKs	[37]
PC-9, H1975	Gefitinib	Sensitive	Cyclins-CDKs	[39]
PC-9	Gefitinib	Sensitive	P53	[38]
A549	Cisplatin	Resistance	miR-17, miR-92, RAD21	[40]
A549	cisplatin	Resistance	P53	[41]
A549	Chemoresistance	Resistance	Nrf-2	[42]
CDK: cyclin-dependent protein; RAD21: Rad21 homolog (S. pombe).				

P21还影响肺癌细胞靶向治疗药物的敏感性。吉非替尼是一种表皮生长因子受体酪氨酸激酶抑制剂，能促进肿瘤细胞凋亡。2011年Zhao等^[37]^发现吉非替尼能通过P53非依赖途径诱导PC-9细胞中P21的表达，同时CDK2/4和cyclin D1/E表达水平降低，细胞周期阻滞在G_1_期，抑制细胞增殖。随后发现吉非替尼耐药的PC-9细胞中药物诱导P21的表达或直接使P21过表达均能增加细胞对吉非替尼的敏感性，这说明P21参与了PC-9细胞对吉非替尼的敏感性。此外，2015年Zhu等^[38]^研究发现，在吉非替尼耐药的PC-9细胞中同时使用白芦藜醇和吉非替尼，能激活P53/P21通路，诱导细胞周期阻滞在G_2_期/M期和细胞衰老，从而增强肺癌细胞对吉非替尼的敏感性。2017年Wang等^[39]^研究发现，与对吉非替尼敏感的PC-9细胞相比，耐药细胞中P21蛋白水平明显下降，通过诱导P21的表达，能降低CDK2/4和cyclin D1/E的活性或表达水平，导致细胞周期阻滞在G_1_期，从而逆转吉非替尼耐药。这说明升高P21表达水平可以增加耐药细胞对吉非替尼的敏感性。然而，也有相反观点，高表达P21能介导肺癌耐药。2015年Zhao等^[40]^发现，P21表达水平升高使细胞周期阻滞在G_1_期，抑制DNA的合成，再加上RAD21高表达增强了DNA的修复作用，抑制了顺铂介导的DNA损伤，从而对顺铂产生耐受。2018年Guo等^[41]^发现，A549细胞在低氧条件下能通过使P53表达上调从而激活P21的表达，使细胞周期阻滞在G_1_期/G_0_期，但此时却增加了A549细胞对顺铂的耐受，其潜在的原因可能是细胞周期阻滞，导致非增殖状态的细胞增加，减小了顺铂对A549细胞的作用。有研究^[42]^发现，Nrf-2可以通过非依赖P53途径直接与*p21*启动子结合，激活*p21*的表达以促进A549细胞在H_2_O_2_诱导的氧化应激下存活。由于肿瘤细胞的活性氧水平升高，同时抗氧化酶水平升高，使它们能抵抗细胞毒性化疗，因此认为Nrf-2/P21途径可能是介导肺癌细胞耐药的原因之一。

P21还在肺癌细胞对放疗的敏感性中扮演重要角色。P21表达下降或过多降解均会导致肺癌细胞耐辐射，上调P21的表达能增加非小细胞肺癌对放疗的敏感性^[44, 45]^。此外，研究^[46]^发现通过激活Akt/mTOR通路靶向P21能促进细胞增殖和诱导耐辐射。

## 5 总结与展望

肺癌耐药机制复杂，大部分体内外实验表明，P21阳性表达是提示肺癌良好预后的标志，P21缺乏是导致肺癌细胞耐药、耐辐射的重要原因之一，其主要机制是P21诱导细胞周期阻滞、细胞衰老和凋亡，从而逆转耐药。但也有研究者^[46]^提出，在肺癌耐药细胞中P21表达升高使细胞周期阻滞从而介导耐药表型。不同的研究结果可能是由于不同实验室使用的细胞类型或细胞状态不一所致。

由于P21在肺癌增殖中的双重作用，P21的表达水平及其在细胞内的定位有望作为肺癌预后的指标，但是调控P21的基因较多，特异性并不高，需要联合其他指标。同时，P21有望作为逆转肺癌耐药和治疗的潜在靶点^[47]^，结合*p53*的状态，在因P21缺乏而导致的耐药和预后不良相关的细胞中应用P21诱导药物，在P21过表达导致的耐药细胞中使用P21抑制剂有望作为肺癌治疗的新方法。我们所面临的挑战是如何能抑制P21介导的促癌和耐药活性，但又不影响其发挥抑癌活性。深入研究P21与肺癌耐药的关系，更好地理解P21在各种情况下的作用机制，有助于解决临床肺癌耐药问题，为临床逆转肺癌耐药和肺癌治疗提供新思路。
